# eUnaG: a new ligand-inducible fluorescent reporter to detect drug transporter activity in live cells

**DOI:** 10.1038/srep41619

**Published:** 2017-02-08

**Authors:** Johannes T.-H. Yeh, Kwangho Nam, Joshua T.-H. Yeh, Norbert Perrimon

**Affiliations:** 1 Department of Genetics, Harvard Medical School, Boston, MA, 02115, USA.; 2 Department of Chemistry and Biochemistry, University of Texas at Arlington, Arlington, TX, 76019-0065, USA; 3 Department of Chemistry, Umeå University, Umeå, SE-901 87, Sweden; 4 Institute of Mathematics, National Taiwan University, Taipei, 10617 Taiwan; 5 Howard Hughes Medical Institute, Harvard Medical School, Boston, MA, 02115, USA

## Abstract

The absorption, distribution, metabolism and excretion (ADME) of metabolites and toxic organic solutes are orchestrated by the ATP-binding cassette (ABC) transporters and the organic solute carrier family (SLC) proteins. A large number of ABC and SLC transpoters exist; however, only a small number have been well characterized. To facilitate the analysis of these transporters, which is important for drug safety and physiological studies, we developed a sensitive genetically encoded bilirubin (BR)-inducible fluorescence sensor (eUnaG) to detect transporter-coupled influx/efflux of organic compounds. This sensor can be used in live cells to measure transporter activity, as excretion of BR depends on ABC and SLC transporters. Applying eUnaG in functional RNAi screens, we characterize *l(2)03659* as a *Drosophila* multidrug resistant-associated ABC transporter.

The absorption, distribution, metabolism and excretion (ADME) of metabolites and organic compounds within an organism are crucial to maintain physiological homeostasis. Two major families of membrane transporters, the ATP-binding cassette transporters (ABC-type transporters) and the solute carrier family (SLC) transporters, are responsible for depositing metabolites and drug molecules into the circulation and excretory systems^1^,^2^. Clinical and pharmacology studies have demonstrated that ABC and SLC transporters are the major determinant factors for drug susceptibility and pharmacokinetics. Importantly, over 90% of metastatic cancers that fail to respond to chemotherapy have been found to have acquired extra copies or high expression of the multi drug resistance-associated ABC transporters^3^. In addition to gene amplification, SNP variants of the SLC or ABC transporters have been implicated with different drug susceptibilities due to altered transporter activities^4^,^5^. Thus, there is increasing interest in the functional and structural characterization of these transporters to better understand chemo-resistance, drug interactions and pharmacokinetics^6^,^7^. However, methods that facilitate rapid cell-based functional and structural studies of these transporters are lacking.

Currently, the most commonly used methods for measuring ABC or SLC transporter activities involve tedious procedures that require the generation and fractionation of artificial transporter containing vesicles derived from disrupted cells^8^ or reconstituted from synthetic lipid vesicles^9^. In order to measure transport activities, a substrate molecule has to be labeled, with radioactive isotope (H3^+^) or chemically conjugated with fluorophores^10^,^11^, to generate the probes. These probes can be difficult to synthesize. Moreover, as these assays are often performed in reconstituted vesicles, they do not allow measurements of transporters activity in live cells, as would be useful for high-throughput functional screens. On the other hand, although analytical chemistry methods such as mass spectrometry can provide accurate measurements directly from biological samples, they cannot be used for live cell-based screens.

To address these issues, we decided to develop a genetically encoded and tractable protein reporter that detects transport of organic compounds. Ligand-inducible and switchable fluorescent sensors, whose signals are turned on only when the analyte (ligand) is present, have many advantages: (1) the signal is ligand-dependent and specific; (2) unlike conventional substrate labeling methods the ligand-inducible reporter requires no washing step to remove unbound fluorophores or extra enzymatic reagents for quantification, further increasing throughput and reducing cost; and (3) the reporter system is genetically encoded and can be easily integrated into various cell types and animal models.

The sensor we engineered is based on the genetically encoded bilirubin (BR)-inducible fluorescence protein UnaG. UnaG, originally identified in the muscle of the Japanese eel, is a cytosolic BR-inducible green fluorescence protein that only fluoresces when the apo-form UnaG is bound with BR^12^. BR is a natural metabolite produced from heme catabolism^13^,^14^. Heme, one of the most abundant organic molecules in the body, is involved in oxygen carriage in red blood cells and also acts as a co-factor for many enzymes such as the large detoxifiying P450 cytochrome family in the liver^14^. Owing to heme abundance, BR itself is also abundant and needs to be efficiently removed from the body as it is toxic to cells. In vertebrates, excretion of BR is mostly mediated by the ABC transporters MRP1-4 and MDR, as well as the SLC transporters OATP1A (Slco1A) and OATP1B (Slco1B).

Based on the observation that MRPs/MDR ABC transporters and OATP transporters recognize a broad spectrum of substrates^7^,^15^, including BR, bile acids and most drug compounds, we reasoned that UnaG could be used as a sensor for rapid assays to reflect the overall activities of ABC and OATP transporters in live cells. We therefore decided to engineer a form of UnaG that could be used as a probe for measuring transporter activities in live cells.

## Results

### Engineering of eUnaG, an enhanced bilirubin (BR)-inducible, GFP-like protein

**Figure 1:**
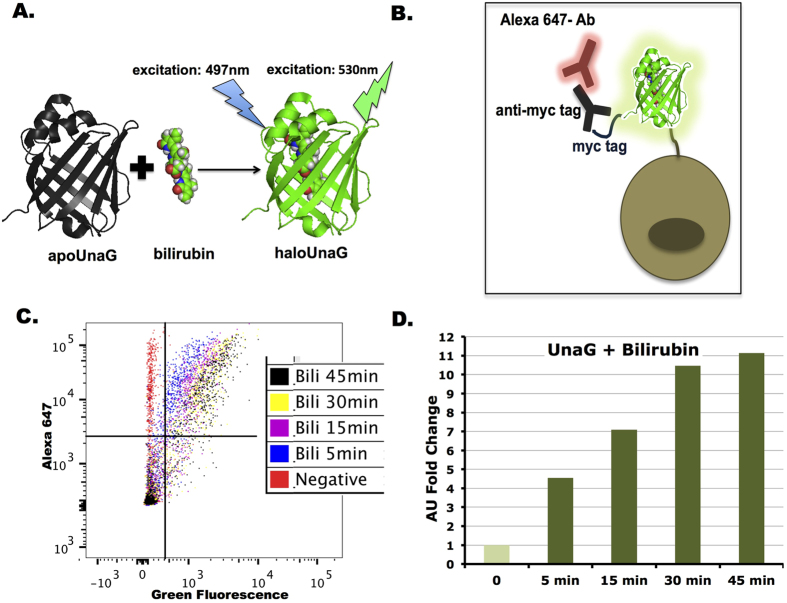
The BR-inducible fluorescence protein UnaG. (**A**) UnaG has a ligand inducible, GFP like fluorescence, when excited at 497 nm upon BR binding. (**B**) Schematic of yeast surface-displayed myc-tagged UnaG for FACS assay. The anti-myc tag/Alexa 647 antibodies were used to normalize UnaG level. (**C**) Time course of BR-inducible UnaG florescence by FACS (BR = 1000 nM). (**D**) Normalized UnaG fluorescence values from (**C**).

BR serves as the fluorochrome in the active holo-UnaG^12^ (Fig. 1A). To assess whether the BR-induced fluorescence signal from UnaG is suitable for quantitative assays in live cells, we constructed a cell surface expression system to display UnaG on the yeast cell surface, incubated the cells with BR, then subjected the cells to FACS analysis (Fig. 1B). The BR-inducible fluorescence signal could be detected as early as 5 minutes after the addition of BR, with increased brightness after 30 minutes (Fig. 1C,D). When UnaG-displaying cells were incubated at 4 °C, cell surface UnaG was still able to form BR-bound fluorescent holo-UnaG, although it took longer to fluoresce and the fluorescent signal was weaker (Fig. S2), likely due to slower binding. These results demonstrate that UnaG can function at different temperatures and has a fast fluorophore association.

**Figure 2:**
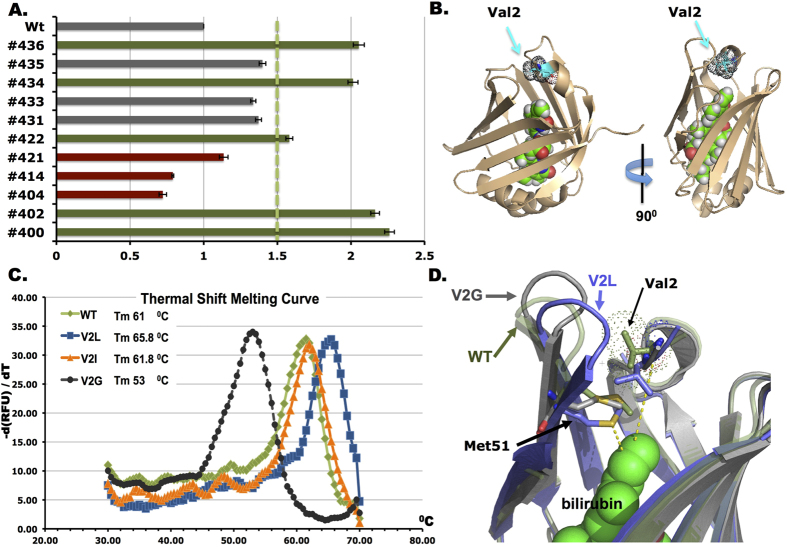
Characterization of enhanced UnaG variants. (**A**) Quantification of BR-induced fluorescence intensity of UnaG variants. (Green color indicates >50% brightness improvement, Red indicates weakened mutants). (**B**) Crystal structure of holo-UnaG (PDB 4I3B). In this complex, the BR fluorophore (shown in spheres) is engulfed inside the UnaG scaffold. The Val 2 residue is indicated in blue. (**C**) BR-induced fluorescence was monitored upon thermal denaturation of holo-UnaG. V2L has the highest thermal stability. (**D**) Average structures of UnaG between the wild-type (green) and V2L (blue) or V2G mutants (gray), determined by the molecular dynamics (MD) simulations. Residues Val2 and Met51 are shown in stick and BR in van der Waals sphere.

**Figure 3:**
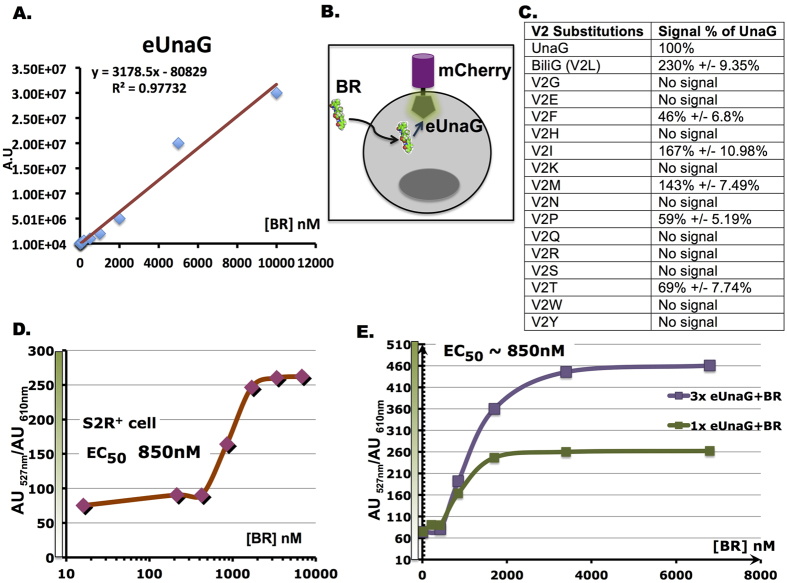
Monitoring cellular BR level using the eUnaG sensor in cultured cells. (**A**) BR dose-response curve with purified eUnaG shows a predictable linear trendline. (**B**) Design of the mCherry-eUnaG reporter for cell-based assays. (**C**) Quantitative fluorescence signal comparison of Val 2 subsitution variants. (**D**) BR dose-response curve of mCherry-eUnaG signal in S2R + cell line. ([BR] = 15 nM, 212.5 nM, 425 nM, 850 nM, 1700 nM, 3400 nM, 6800 nM). (**E**) BR dose-response curve of S2R + cells expressing different levels of mCherry-eUnaG (1X vs. 3X reporter transfected). Despite the increase in signal intensity and dynamic range, the EC50 of from both groups was not affected. (mCherry-eUnaG expression levels are shown in the box to the right).

As a brighter fluorescence signal would be advantageous to most bioimagining and live cell analyses, we decided to engineer brighter UnaG mutants with increased detection sensitivity. By using error-prone PCR mutagenesis^16^, we generated a UnaG mutant library to be displayed on the yeast cell surface. Mutant cells fom this library associated with stronger BR-inducible mean fluorescence intensities were isolated by FACS. After three rounds of FACS selection and enrichment, we isolated individual clones with increased BR-induced fluorescence intensities as compared to wild type UnaG (Fig. 2A). Interestingly, sequence alignment of the selected UnaG variants revealed a common highly conserved Valine to Leucine (V2L) mutation in all the mutant clones (Fig. S3), suggesting that the V2L mutation alone is responsible for enhanced BR-induced fluorescence. To test this hypothesis, we substituted Val 2 with various amino acids and tested them in yeast. Strikingly, only V2L showed the most significant improvement of BR-induced fluorescence. This finding was also observed in insect cell line S2R experiment, with V2L the most enhanced substitution whereas other substitutions (V2E, V2H, V2K, V2N, V2Q, V2R, V2S) were deleterious to protein fluorescence (see Fig. 3C for details). This result was interesting because in the X-ray crystal structure of UnaG in complex with BR (Fig. 2B) Val 2 does not form a direct interaction with BR^12^. In contrast, in the case of an improved GFP protein, the optimized mutant residues (i.e. F64L & S65T)^17^ overlap or are adjacent to the fluorophore center, i.e. Ser 65, Tyr 66, and Gly 67. One possible explanation for the enhanced UnaG is that substitution of Valine by a larger hydrophobic Leucine results in a more stable protein scaffold, possibly achieved by providing a further stabilzed interaction between the fluorophore ligand (BR) and the residues within the hydrophobic core of UnaG scaffold. To test this hypothesis, we used purified proteins to assess whether the V2L UnaG-BR complex is more stable than the wild type UnaG-BR complex. Utilizing BR-dependent fluorescence, we used a qPCR thermocycler to measure the melting temperature (Tm) of proteins that had already been complexed with BR. Strikingly, the V2L holo-UnaG has higher thermal stability (Tm 65.8 °C) than wild type holo-UnaG (Tm 60 °C). By contrast, substitution of Valine 2 by Glycine results in substantial decrease of UnaG’s thermal stability, indicating the importance of having a hydrophobic residue at residue 2 for proper protein scaffolding (Fig. 2C). However, introducing a hydrophobic Isoleucine instead of a Leucine at residue 2 only slightly increased UnaG thermal stability. The effects of these mutations on the local structure of residue 2 were examined by molecular dynamics (MD) simulations. The simulations carried out up to 100 ns for each mutatant revealed the importance of interactions of residues 2 in the local structure, in particular, the orientation of a loop in contact with residue 2 (Fig. 2D). Our current hypothesis is that the V2L mutation pushes Met 51 toward BR to increase its contact interaction with BR, via stabilization of the loop that is connected to Met 51 (Fig. S4). This result correlates well with the changes of BR-inducible fluorescence found in the FACS assay and thermal stability of these mutants. Taken together, our directed evolution-based selection strategy identified the V2L substitution as a better fit for enhanced thermal stability with increased BR-induced fluorescence.

### Assaying BR transport and equilibrium by eUnaG sensor in live cells

Using purified eUnaG, we found that the BR-inducible fluorescence signal linearly correlated with BR concentration (Figs 3A, S5), indicating that the sensor provides quantitative comparison of BR concentration inside the cells. Next, we designed a sensor comprised of an mCherry-eUnaG fusion protein (Figs 3B, S6) for live cell assays. In this construct the mCherry signal is used to normalize the expression level of the sensor among different samples, whereas the green fluorescence signal reflects BR concentration inside the cells.

We next tested whether we could assay transporter activities using cellular BR levels as a readout in *Drosophila* S2R + cells. We first tested whether eUnaG (V2L substitution) in the insect expression system remained the most performant substitution among the previous variants tested previously. As shown in Fig. 3C, eUnaG still showed the best fluorescence signal, in a good agreement with our Tm and MD simulation experiments. Next, we tested the use of eUnaG for ADME assays in S2R + cells. Through serial titration of BR spiked into the culture medium, we observed that the mean fluorescence intensity ratio of eUnaG to mCherry increased as the amount of BR added increased (Fig. 3D). In addition, when the expression level of the sensor was increased, both the fluorescence signal and the dynamic range were increased, and the system was seemingly more sensitive to low levels of BR (Figs 3E, S7). However, despite the increased dynamic range, the EC_50_ for reporter signal saturation remained the same, suggesting that the equilibrium of BR influx/efflux in the cells was saturated at about 3 μM of BR within 15 minutes (Fig. S8). Judging from the saturatable BR uptake curve observed for S2R + cells, BR permeability is likely largely determined by active transport rather than passive diffusion- otherwise one would expect to see a constantly increasing reporter signal over increasing BR dose instead of a response curve reaching a plateau. Interestingly, testing of a different *Drosophila* cell line, Kc167, revealed a weaker cellular uptake pattern (Fig. S9), presumably due to differential expression of the transporters^18^ in this cell line. These observations suggest that the mCherry-eUnaG sensor is suitable for testing the activities of organic compound transporters, as the equilibrium of BR would depend on the expression ratio or corresponding activities of influx/eflux transporters. It is also worth mentioning that because of the ligand dependency, the BR-induced fluorescent reporter assay requires no extra washing procedures, which are usually necessary for conventional fluorescent dye-labeled probes. This feature presents a significant advantage as it provides a simplified and time saving method for transporter assays, thus facilitating high-throughput studies.

### Using eUnaG to assay transporter-associated drug interactions

**Figure 4:**
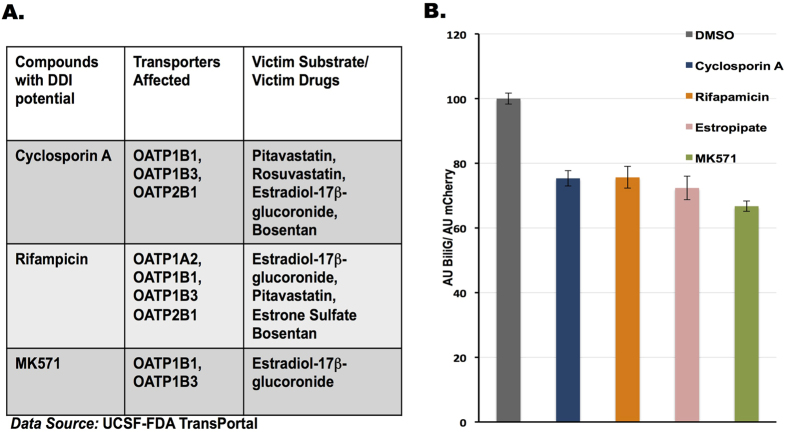
eUnaG sensor for drug interaction assays. (**A**) Drug interaction information for compounds tested in this study. (**B**) DDI assay. S2R + cells were pretreated with Cyclosporine A, Rifapamicin, Estropitate (50 μM each) or DMSO for 1 hour before adding BR (1700 nM). The eUnaG signal was measured 1 hour after BR incubation.

In mammals the clearance of metabolites and xenobiotics, including BR, is mostly mediated by hepatic and renal excretion. This process involves the compounds being taken up by the liver or kidney through the OATP transporters (OATPs)^19^, followed by excretion through the ABC transporters MRP or MDR^1^. Both OATP and MRP/MDR transporter activities are essential pharmacokinetic effectors. In the clinic, many drugs have been shown to affect the ADME of other drugs by attenuating the activities of OATP transporters when administrated together in patients, a phenomenon known as drug-drug interaction (DDI)^20^ (Fig. 4A). Conversely, DDI can cause toxicity whereby one drug inhibits the ADME of other toxic compounds in the body. Despite the large diversity of OATP and ABC transporters in animals, many of these transporters across species share broad and overlapping substrates and possibly will have similar DDI susceptibility. With this in mind, we next asked if the eUnaG reporter could be used to test compound DDI effects in live cells. In S2R + reporter cells treated with compounds known to have DDI effects, Cyclosporine A, Rifampacin, Estropipate and MK 571^20^,^21^, we observed a ~25–35% reduction in eUnaG reporter activity (Fig. 4B), demonstrating that the eUnaG based method can reflect the tendency of a compound to cause DDI via some of the OATP transporters. This finding demonstrates that the eUnaG reporter can be used as a simple and quick screen assay for compounds that can potentially interfere with drug or metabolite transporters.

### eUnaG as a probe to assay mitochondrial-stress induced ABC transporter activity in live cells

**Figure 5:**
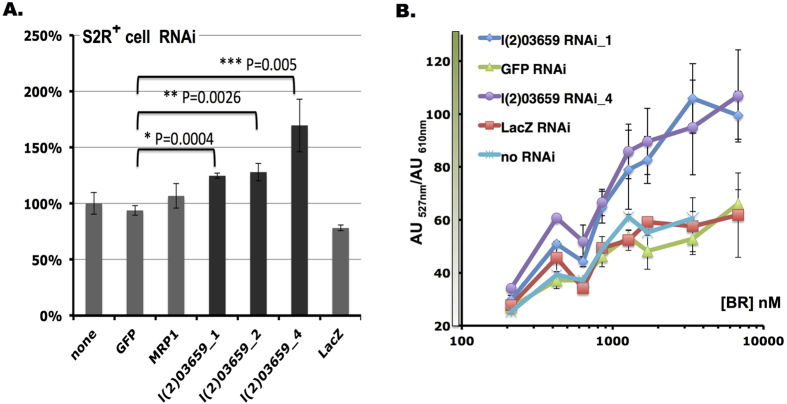
*l(2)03659* encodes a major *Drosophila* ABC transporter for BR. (**A**) RNAi of transporters revealed that *l(2)03659* is involved in BR efflux. GFP RNAi was used as a control. (**B**) BR dose-response curve of S2R + cells treated with *l(2)03659* RNAi. *GFP* and *lacZ* RNAi were used as controls.

Having demonstrated that the eUnaG-mCherry sensor is a useful reporter for transporter activities, we used eUnaG in a functional screen to identify key transporters that control active BR transport in S2R + cells. We used RNAi to knock down a panel of genes encoding putative OATP and ABC transporters, and used high-throughput FACS to quantitate the effects of knockdown on the reporter signal. Strikingly, RNAi against the *Drosophila* ABC transporter *l(2)03659* significantly increased the reporter signal (Fig. 5A). A 25~50% increased intracellular BR level was observed with three independent *l(2)03659* RNAi constructs (Fig. 5A; Fig. S10). Further analysis revealed that *l(2)03659* encodes a member of the ABC transporter super family and is homologous to human multidrug resistance-associated protein MRP4/ABCC4^22^ (http://www.genenames.org/cgi-bin/gene_symbol_report?hgnc_id=HGNC:55), suggesting that the ABC transporter ecoded by *l(2)03659* plays a major role in pumping BR outside the cell. Further, we performed a BR titration experiment measuring the eUnaG signal of cells treated with RNAi reagents targeting *l(2)03659*. The two different RNAi constructs targeting the gene both showed the same increased BR-dependent signal (Fig. 5B), consistent with *l(2)03659* acting as a *bona fide* regulator of BR transport.

**Figure 6:**
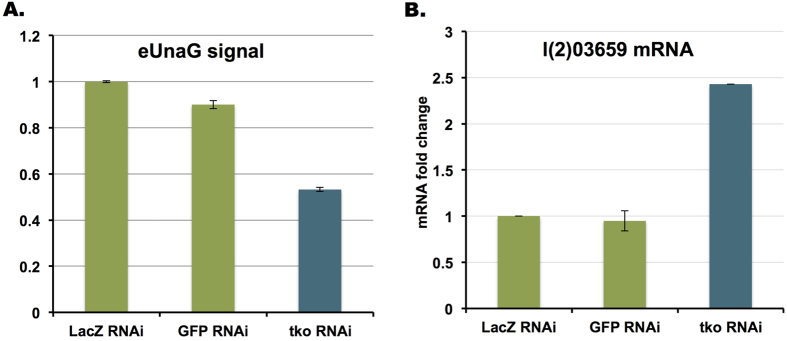
Elevated BR export in response to cellular mitochondria stress. (**A**) eUnaG reporter signal is significantly decreased in S2R + cells treated with *tko* RNAi. *GFP* and *lacZ* RNAi are used as controls. (**B**) qRT-PCR showing elevated *l(2)03659* mRNA expression in *tko* RNAi cells, but not in *GFP* and *lacZ* RNAi controls.

Interestingly, a recent microarray experiment^23^ has indicated that *l(2)03659* is up-regulated in flies mutant for the *Drosophila* mitoribosomal protein S12 (*tko*^25*t*^), suggesting that *l(2)03659* is a mitochondrial stress-responsive gene. To test whether mitochondrial stress can result in elevated xenobiotic transport, we treated S2R + cells with *tko* RNAi and used the eUnaG sensor as a readout. As shown in Fig. 6A, the eUnaG reporter signal was significantly reduced in cells treated with *tko* RNAi but not control RNAi. Consistent with the eUnaG reporter assay, *l(2)03659* expression levels were up-regulated in *tko* knockdown cells as well (Fig. 6B), suggesting that *l(2)03659* is a mitochondrial stress responsive gene activated by cellular mitoribosomal protein S12 insufficiency. Altogether, these results suggest that elevated clearance of xenobiotics or metabolites might be an essential mitochondrial stress-relief mechanism.

## Discussion

Fluorescent proteins are crucial tools for biological studies. Although a wide array of GFP derivative variants have been developed, easy-to-use, ligand-induced, fluorescent proteins are rare. The fatty acid binding protein UnaG is one of the very few ligand-inducible fluorescent proteins characterized so far^12^. In this study, we show for the first time that UnaG can be used for cell-based functional studies of xenobiotics and metabolite transporters, and characterize a UnaG variant, eUnaG, with increased fluorescence and thermostability. We have further extended the study of Kumagai *et al*.^12^ on UnaG by demonstrating that the eUnaG-based sensor can reflect BR uptake in live cells. This finding led us to test the use of eUnaG as a sensor for functional transporter studies.

In the clinic, serum BR levels can serve as an indicator of hepatic function^24^, as BR clearance is largely mediated by hepatic uptake and excretion. Dysregulation of BR clearance causes syndromes such as jaundice and Dubin-Johnson Syndrome^25^. OATP1A and OATP1B are the two transporters responsible for BR hepatic uptake in humans. These transporters are also known to be essential transporters for drug and waste removal by excretory organs. Indeed, transporters in general have wide substrate spectrums, with the ability to transport a large variety of metabolites and compounds in the body. Many ABC transporters, for example, pump out compounds naturally produced by cells and are implicated in drug resistance. These properties make eUnaG a good general tool for transporter assays, as it is likely that many transporters of interest can recognize BR as a substrate. We also showed that eUnaG can be used as a simple functional readout for assaying potential DDIs. Genetic studies have shown that SNPs in SLC/OATPs can have significant impacts on drug efficacy, DDI, and the DDI-associated toxicity response^5^,^26^. Determining functional SNPs from different populations has been valuable for drug development. We expect that eUnaG will provide a rapid and cost-effective tool for cell based functional characterization of SNPs associated with transporters.

Using eUnaG as a reporter, we identified the ABC transporter encoded by *l(2)03659* as a key regulator of BR export in *Drosophila* cells. We further observed that cellular mitochondria stress induced by knockdown of the mitoribosomal protein S12^27^ causes up regulation of *l(2)03659,* which may ultimately lead to increased export of metabolites and xenobiotics. Although it remains to be elucidated why the *l(2)03659* ABC transporter is required in response to mitochondrial stress, one possible function of *l(2)03659* may be to prevent the accumulation of molecules toxic to the nuclear and mitochondrial genomes^23^.

In a recent review^28^, Cesar-Razquin *et al*. described the need for wider research on transporters, in particular SLC class transporters, and argued that SLC transporters are understudied in biology. A limitation in the field has been the methodologies used for functional characterization of these transporters in the context of cell biology, which relied on reconstituted or synthetic vesicles. The availability of tools such as the eUnaG live cell assay we describe should provide a straightforward approach, for example for functional genomic screens or small molecule screens for ABC transporter inhibitors. Potentially, the concept of using ligand-inducible fluorescent proteins could be further generalized to design or engineer synthetic ligand/scaffold pairs as new set of probes to monitor different classes of transporters.

## Methods

### UnaG constructs

The UnaG cDNA was designed based on the amino acid sequence with codons optimized for bacteria, yeast and mammalian expression (see Supplementary Information S1 for the cDNA sequence). DNA oligos were synthesized by IDT DNA Technology Inc based on the designed sequence. Depending on the destination expression plasmids to be used, UnaG ORF was amplified by PCR, followed by standard restriction enzyme digestion and ligation into individual destination vectors such as pET21a, pMAL C2X, pMT-V5His or pcDNA3.1.

### Library screening for eUnaG

We employed error-prone mutagenesis PCR amplification^29^ of UnaG ORF to generate a UnaG mutant library. For error-prone PCR condition, standard Taq polymerase PCR reaction setup was used except that the dGTP was reduced from 200 μM to 40 μM, in addition to supplement 480 μM MnSO_4_ in the reaction buffer. 40 amplification cycles were performed. The resulting UnaG amplicons harboring random mutations were then cloned into the pYD expression vector and transformed into yeast cells. Next, library cells were amplified in Tryptophan dropout medium. For FACS selection, 10^8^ yeast cells incubated with 700 nM BR were sorted. The wild type UnaG green fluorescence intensity was used as a cut off. Only cell populations having stronger BR-inducible green fluorescence signal were collected. Collected cells were then amplified to repeat another two rounds of cell sorting. Afterwards, cells were plated on agar plates and the individual colonies were picked up to confirm the enhanced BR-induced fluorescence by FACS.

### Protein purification

As UnaG-His6 or eUnaG-His6 gave very low protein yields in our hands, we generated MBP fusion constructs (pMAL C2X vector, New England Biolabs) containing TEV cleavage site between MBP and UnaG/eUnaG for all the UnaG variants to increase the protein expression levels. BL21 (DE3) *E. coli* was used for protein expression following standard procedures. In brief, cells were culture at 37 °C until OD_600_ reached 0.8~1. IPTG induction was done at 25 °C overnight. Cell pellets were collected, resuspended in 20 mM Tris, pH8.0, 300 mM NaCl, sonicated and centrifuged to collect the soluble fractions. For affinity purification, total soluble fractions were incubated with amylose beads at 4 °C. Amylose beads were then washed with 100X volume of wash buffer 20 mM Tris, pH8.0, 300 mM NaCl and the UnaG proteins were cleaved from MBP attached to the beads by TEV protease. The TEV protease was later removed with Ni-NTA from the UnaG elution.

### Melting temperature measurement

Purified proteins were incubated with BR for 20 minutes at room temperature. The samples were loaded in a 96-well real time qPCR plate and subjected to gradient thermal denaturing from 27 °C to 70 °C with 0.5 °C increment in a real-time qPCR thermocycler. The green fluorescence signal was measured as an indication of protein folding. The melting curve is derived from the instrument reading with the same routine procedure used for oligonucleotides. Similarly, the melting temperature (Tm) is determined by from the melting curve using the function of –d (RFU)/dT wherein RFU is the fluorescence signal and T is the temperature.

### eUnaG sensors in cell based assays

*Drosophila* S2R + and Kc167 cells were cultured in a 10-cm cell culture plate and transiently transfected with 10 ug pMT mCherry-eUnaG plasmid. To induce mCherry-eUnaG expression, 500 uM CuSO_4_ was added to the culture medium 24 hours after plasmid transfection. BR-induced fluorescence can be detected within 12 hours after CuSO_4_ induction. For DDI experiments, CuSO_4_ cells were treated with indicated amount of compounds or vehicle (DMSO) alone for 1 hour prior to BR addition. After incubation with BR for another hour, cells were kept on ice followed by FACS analysis. For transporter RNAi experiments, 24 hours after transfection, cells were switched to serum free medium and split into 12-well plates (2 × 10^6^ cells/well density), 5 μg of dsRNA was added. 30 minutes after incubation, serum-containing medium was added and the cells were cultured in the incubator for 3 days to allow the knockdown of target transcripts. Cells were then induced with CuSO_4_ for 24 hours before BR treatment.

For measuring cellular BR level, reporter cells were incubated with indicated amounts of BR for a period described in the main text. Cells were then resuspended for FACS analysis. For quantification the mean green fluorescence signal was normalized with the mean mCherry signal.

## Additional Information

**How to cite this article**: Yeh, J. T.-H. *et al*. eUnaG: a new ligand-inducible fluorescent reporter to detect drug transporter activity in live cells. *Sci. Rep.* **7**, 41619; doi: 10.1038/srep41619 (2017).

**Publisher's note:** Springer Nature remains neutral with regard to jurisdictional claims in published maps and institutional affiliations.

## Supplementary Material

Supplementary Information
