# Supplementary material for: eUnaG: a new ligand-inducible fluorescent reporter to detect drug transporter activity in live cells
Source: Sci Rep. 2017 Feb 8;7:41619. doi: 10.1038/srep41619 (PMC5296874; doi:10.1038/srep41619)
Supplement: Supplementary Information [file srep41619-s1.pdf]

**Supplementary Materials**

# eUnaG: a new ligand-inducible fluorescent reporter to detect drug transporter activity in live cells

Johannes T.-H. Yeh[1,6], Kwangho Nam[3,4], Joshua T.-H. Yeh[5], Norbert Perrimon[1,2]

[1]Department of Genetics, Harvard Medical School, Boston, MA, 02115, USA

[2]Howard Hughes Medical Institute, Harvard Medical School, Boston, MA, 02115, USA

[3]Department of Chemistry and Biochemistry, University of Texas at Arlington, Arlington, TX, 76019-0065

[4]Department of Chemistry, Umeå University, Umeå, SE-901 87, Sweden

[5]National Taiwan University, Institute of Mathematics, Taiwan

[6]Present Address: Cold Spring Harbor Laboratory, Cold Spring Harbor, NY 11724, USA

Correspondence should be addressed to yehjth@gmail.com or perrimon@receptor.med.harvard.edu

## Supplementary Method

### Molecular dynamics (MD) simulations

The systems for molecular dynamics simulation were prepared based on the X-ray crystallographic structure determined by Kumagai *et al.* (PDB ID: 4I3B)[1].The first monomer (chain A), its surrounding waters, and bound bilirubin (BR) were used in the system preparation. For the wild-type system, the structure was used without modification. For the V2L and V2G mutants, Val 2 residue was mutated to the corresponding residue. Protonation states of all ionizable residues were assigned based on the positions of protons of each protonatable residue found in the X-ray structure. Each system was solvated with 64.0 Å rhombic dodecahedron (RHDO) box of waters, followed by removal of waters within 2.5 Å from any protein heavy atom. The systems were then neutralized with 15 $Na^+$ and 14 $Cl^-$ ions. The procedure resulted in, for example, a total of 18,760 atoms for the wild-type system, including 5,482 waters.

Each system was first energy minimized for 6,000 steps with series of constraints and restraints applied to protein heavy atoms, to alleviate steric clashes present in the starting structure. After heating up from 8 K to 298 K over 24 ps, each system was simulated for 100 ns at 298 K. In the simulation, the protein and Na+/Cl- ions were represented by the all-atom CHARMM36 force fields with the CMAP correction[2, 3], and waters by the fixed-geometry TIP3P water model[4]. The bilirubin was described by the CGenFF36 force fields[5]. The particle-mesh Ewald summation method[6] was used for electrostatic interactions with the 11 Å real space cutoff and 60 × 60 × 60 fast Fourier transform (FFT) grids. The van der Waals interactions were truncated at the same cutoff distance with the switching function applied between 9 Å and 11 Å. The simulations were carried out with the leap-frog Verlet algorithm with 2 fs as the integration time and the SHAKE-like algorithms[7, 8] applied to all bonds involving hydrogen atoms. The Langevin thermostat was used to maintain each system's temperature at 298 K. The CHARMM program (version c41a1)[9] and the CHARMM-GUI web interface[10] were used in the system preparation, and the NAMD program (version 2.9)[11] in the production MD, respectively.

**S1. cDNA sequence of UnaG**

```
atggttgaaaagttcgtagggacctggaaaatagcagatagtcacaactttggcgagtat
 M  V  E  K  F  V  G  T  W  K  I  A  D  S  H  N  F  G  E  Y
ttaaaggcaataggcgcaccaaaagaattatccgatggtggagatgccaccacaccaacc
 L  K  A  I  G  A  P  K  E  L  S  D  G  G  D  A  T  T  P  T
ttatacattagtcagaaagacggtgacaaaatgactgtaaagattgaaaacgggccacca
 L  Y  I  S  Q  K  D  G  D  K  M  T  V  K  I  E  N  G  P  P
acattttttagacacgcaagtcaaatttaaattaggcgaagaatttgacgaatttccaagt
 T  F  L  D  T  Q  V  K  F  K  L  G  E  E  F  D  E  F  P  S
gatagaaggaagggtgtgaagtctgtagttaatctagtcggtgagaaattggtttatgtc
 D  R  R  K  G  V  K  S  V  V  N  L  V  G  E  K  L  V  Y  V
caaaaatgggatggtaaggagaccacatatgtgagggaaataaaagacggtaaacttgtg
 Q  K  W  D  G  K  E  T  T  Y  V  R  E  I  K  D  G  K  L  V
gtgactttgacaatgggagacgttgtggctgtaaggagttatagaagggcaaccgaggga
 V  T  L  T  M  G  D  V  V  A  V  R  S  Y  R  R  A  T  E  G
tccactagtccagtgtggtggaattctgcagatatccagcacagtggcgcc
 S  T  S  P  V  W  W  N  S  A  D  I  Q  H  S  G  A
```

**S2. Time course of BR-induced green fluorescence in surfaced-displayed cells by FACS**

(A) FACS analysis of yeast surface displayed UnaG. Cells were incubated with 1700nM BR at room temperature for the indicated amount of time before FACS analysis. UnaG expressing populations are labeled with Alexa 647. Note that the appearance of Alexa $647^{+}$/ Green Fluorescence$^{+}$ cells after 5 minutes of BR incubation.

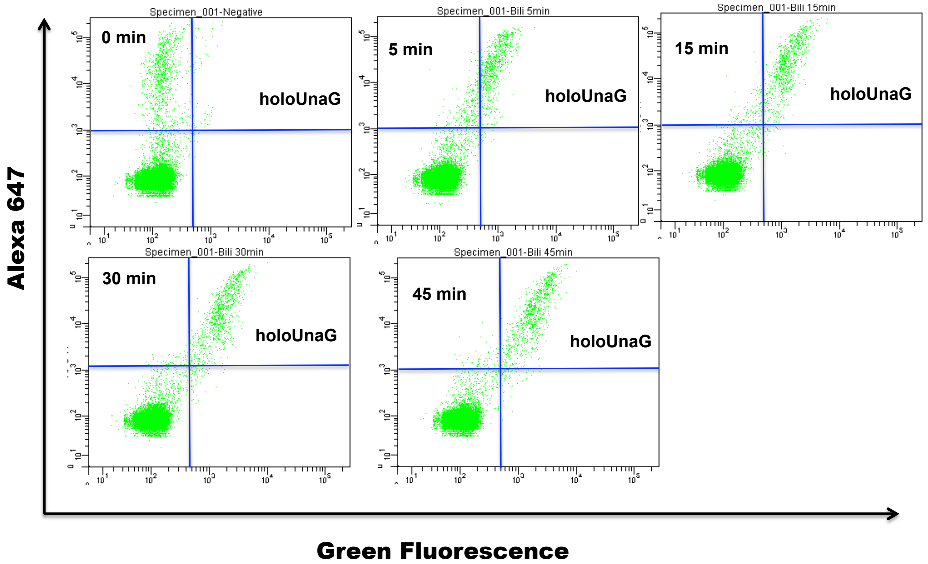


(B) Similar to (A), cells were incubated with 1700nM at $4^{0}$C with indicated amount of time immediately followed by FACS analysis. A very small fraction of Alexa $647^{+}$/Green Fluorescence$^{+}$ cells appeared after 5 minutes of BR incubation and it took a longer period for the majority of Alexa $647^{+}$ cells to turn green.

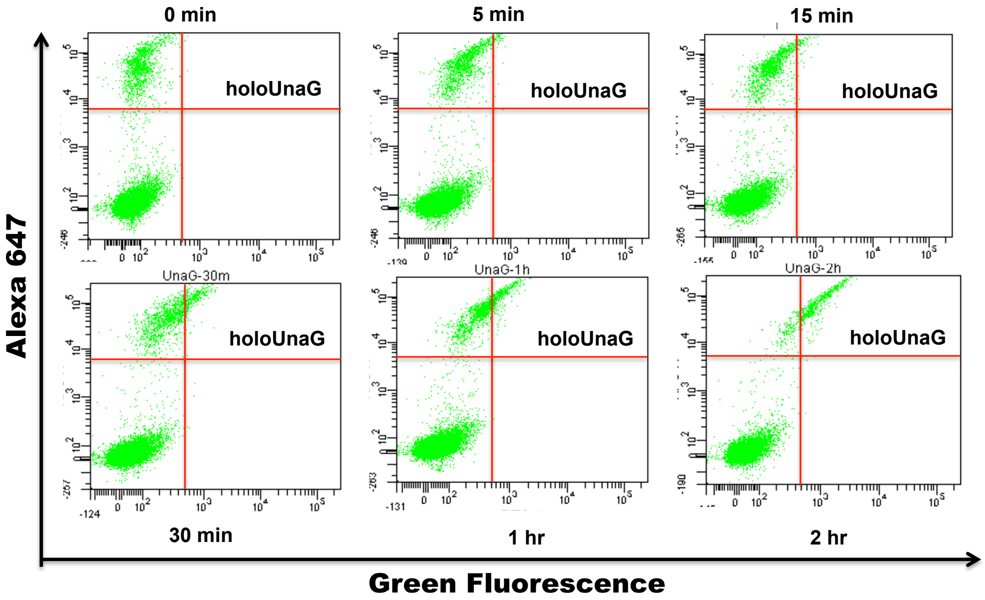

## S3. Sequences of clones with enhanced BR-inducible fluorescence

The highly conserved V2L mutation is highlighted in green. Additional mutations other than V2L are highlighted in cyan.

**Wt UnaG>**
**MVEKFVGTWKIADSHNFGEYLKAIGAPKELSDGGDATTPTLYISQKDGDKMTVKIENGPPTFLDTQ**
**VKFKLGEEFDEFPSDRRKGVKSVVNLVGEKLVYVQKWDGKETTYVREIKDGKLVVTLTMGDVVAVR**
**SYRRATE**
**400(eUnaG)>**
**MLEKFVGTWKIADSHNFGEYLKAIGAPKELSDGGDATTPTLYISQKDGDKMTVKIENGPPTFLDTQ**
**VKFKLGEEFDEFPSDRRKGVKSVVNLVGEKLVYVQKWDGKETTYVREIKDGKLVVTLTMGDVVAVR**
**SYRRATE**
**402>**
**MLEKFVGTWKIADSHNFGEYLKAIGAPKELSDGGDATTPTLYISQKDGDRMTVKIENGPPTFLDTQ**
**VKFKLGEEFDEFPSDRRKGVKSVVNLVGEKLVYVQKWDGKETTHVREIKDGKLVVTLTMGDVVAVR**
**SYRRATE**
**422>**
**MLEKFVGTWKIADSHNFGEYLKAIGAPKELSDGGDATTPTLYISQKDGDKMTVKIENGPPTFLDTQ**
**VKFKLGEEFDEFPSDGRKGVRSVVNLVGEKLVYVQKWGGKETTYVREIKDGKLVVTLTMGDVVAVR**
**SYRRATE**
**431>**
**MLEKFVGTWKIADSHNFGEYLKAIGAPKELSDGGDATTPTLYISQKGGDKMTVKIENGPPTFLDTQ**
**VKFKLGEEFDEFPSDGRKGVKSVVNLVGEKLVYVQKWDGKETTYVREIKDGKLVVTLTMGDVVAVR**
**SYRRATE**
**433>**
**MLEKFVGTWKIADSHNFGEYLKAIGAPKELSDGGDATTPTLYISQKDGDKMTVKIENGPPTFLDTQ**
**VKFKLGEEFDEFPSDGRKGVKSVVNLVGEKLVYVQKWDGKETTYVREIKDGKLVVTLTMGDVVAVR**
**RYRRATE**
**434>**
**MLEKFVGTWKIADSHNFGEYLKAIGAPKELSDGGDATTPTLYISQKDGDEMTVKIENGPPTFLDTQ**
**VKFKLGEEFDEFPSDRRKGVKSVVNLVGEKLVYVQKWDGKETTYVREIKDGKLVVTLTMGDVVAVR**
**SYRRATE**

**S4. Molecular Dynamics (MD) modeling of V2L & V2G mutation**

(A) Comparison of structures between the wild-type (light green) and V2L (light blue) and V2G mutants (gray). Each structure was determined based on the atomic coordinates saved during the 100 ns MD simulations, in which the last 50 ns time frames were used to determine the average structures. The region indicated in the left panel was enlarged in the right panel with a slight rotation of the structure for clear visualization of the structure near the residue 2 and Met 51. Bilirubin (BLR) was shown in green van der Waals sphere. The yellow dotted lines were for the distances from BLR C16 atom to V2L Cα atom and also to Met 51 Cα of the V2L mutant (See below for the change of these distances during MD simulation).

(B) Distance changes during MD simulations. In the upper panel, the distance between Cα of residue 2 and BLR C16 atoms were shown, in which the wild-type was shown in green, V2L in blue, and V2G in black, respectively. In the lower panel, the distance between Met 51 S and BR C16 atoms were shown. The colors are the same as those used in the upper panel. The two distances show that the V2L systems are most stable in terms of their distance fluctuations during MD, while the wild-type has substantial fluctuation during MD. The V2G system showed the longest distances and largest fluctuations. This difference of distance fluctuations are consistent with the structures shown in (A), which show that V2G has the largest opening of the loop connected to Met 51, while V2L has the loop collapsed with Met 51 pushed towards BLR. The wild-type has its loop orientation in between the V2L and V2G mutants.

**A.**

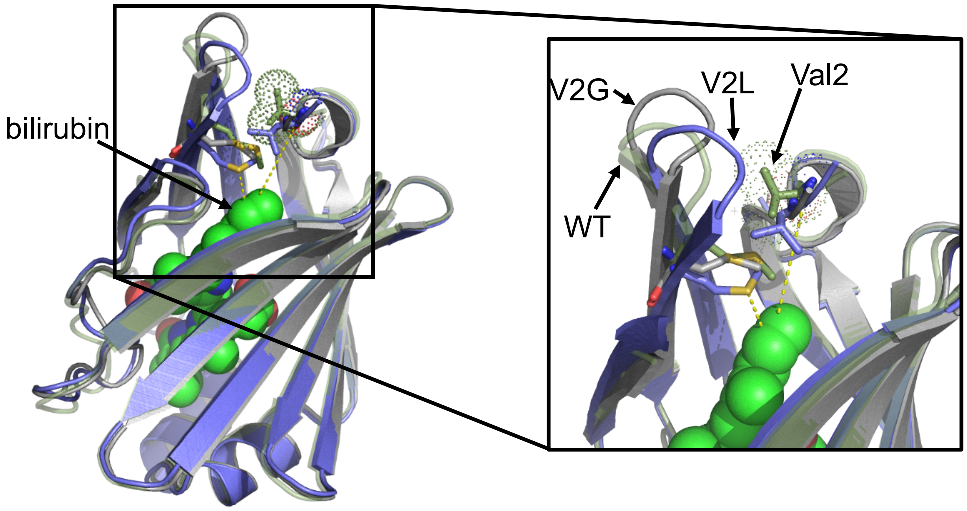


**B.**

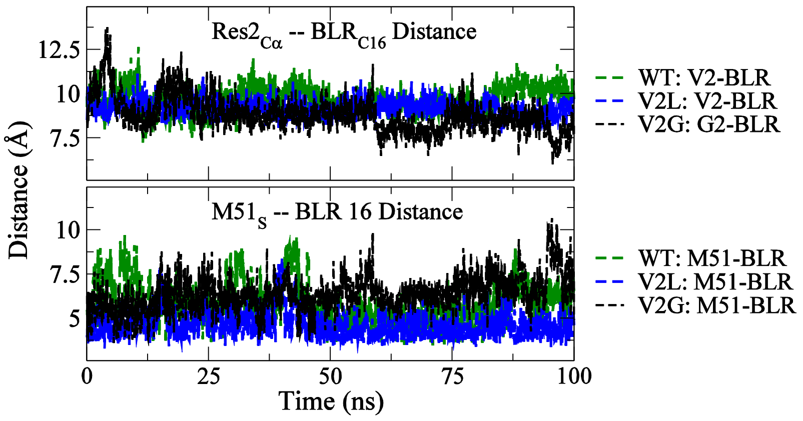

**S5. Fluorescence spectrometry showing BR can compete with 1,8 ANS for eUnaG binding.**

(A) Purified eUnaG or BSA (10 µM) was incubated with various amount of BR. Fluorescence intensity was measured with Ex 498/ Em 530 performed with a fluorescence plate reader (Molecular Devices). Although BSA is also a known BR binding protein[1], BR binding only induce fluorescence with UnaG protein scaffold.

(B) 1,8-ANS (1-anilinonaphthalene- 8-sulfonic acid) is an environment-sensitive dye which is essentially non-fluorescent in solution alone, and only become fluorescent (Ex 350nm/ Em 470nm) when it is bound to hydrophobic surfaces. This property makes it a sensitive indicator for detecting hydrophobic cavities in proteins, albeit promiscuously. This probe has been used to determine hydrophobic ligands binding to receptors such as free fatty acids to fatty acid binding proteins (FABP)[12].

(C) Since UnaG belongs to the FABP superfamily, we tested whether 1,8 ANS can bind to the BR binding pocket. Titrating 1,8-ANS to purified eUnaG (5 µM) generated blue fluorescence (Ex 350nm/ Em 470nm) with good linearity, indicating that the small molecule can bind to the classical FABP family ligand-binding pockets. On the contrary to BR binding, 1,8 ANS binding to BSA (5 µM) showed 1,8 ANS-dependent fluorescence.

(D) To determine if BR can compete for 1,8 ANS binding to eUnaG, various amounts of BR were added to preformed eUnaG or BSA-1,8 ANS complex (5 µM protein incubated with 500nM 1,8 ANS). Blue fluorescence of protein-bound 1,8 ANS were decreased upon increased BR concentration, indicating a replacement of 1,8 ANS by BR.

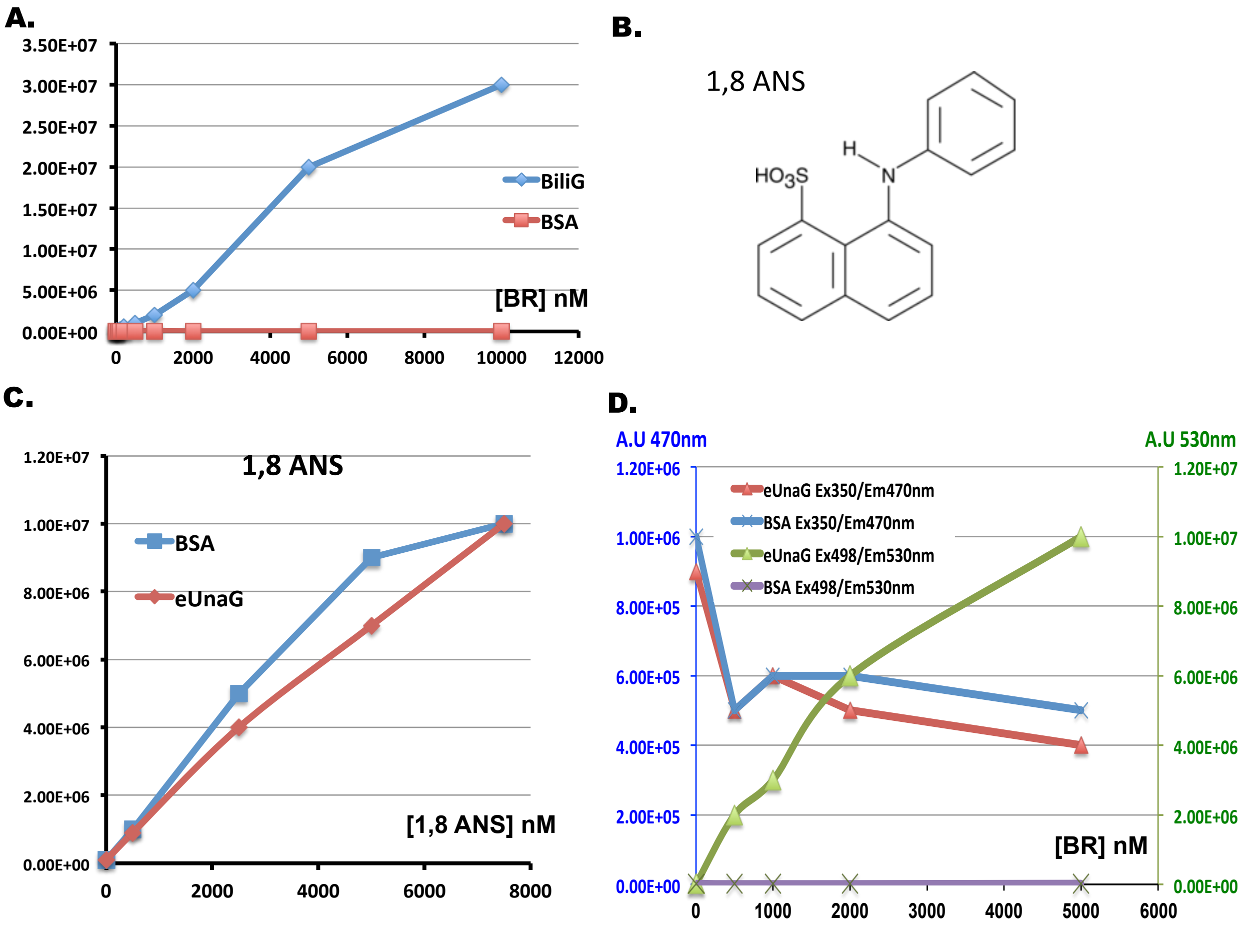

**S6. The design of mCherry-eUnaG sensor for expression in insect cells**

The mCherry-eUnaG sensor is a transmembrane fusion protein composed of an extracellular mCherry domain, a single spanning transmembrane region and a cytosolic eUnaG domain. This construct generates a membrane concentrated strong fluorescence signal in FACS.

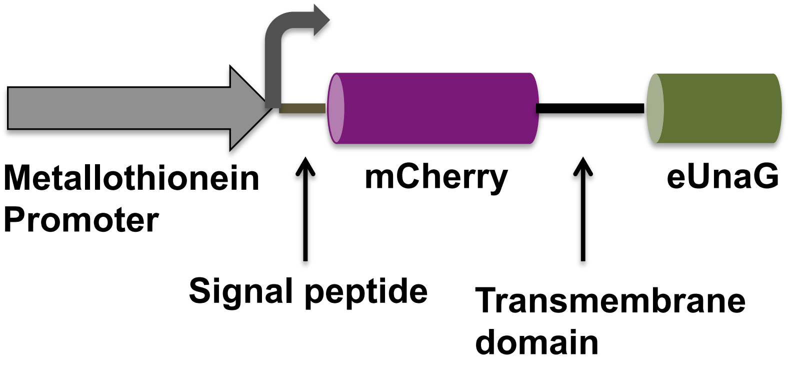


The sequence of the signal peptide is: **MLRLLLALNLFPSIQVTG**

The sequence of the transmembrane domain is: **FWVLVVVGGVLACYSLLVTVAFIIFWV**

**S7. mCherry-eUnaG expression levels quantified by mCherry shown in Fig-3E.**
Using the mCherry-eUnaG fusion construct, the expression level of individual cells can be quantitated by the signal intensity of mCherry.
As described in Fig-3E, cells with higher mCherry-eUnaG expression levels showed increased dynamic range of BR detection, however, the EC50 of BR transport remained the same (850~900 nM).

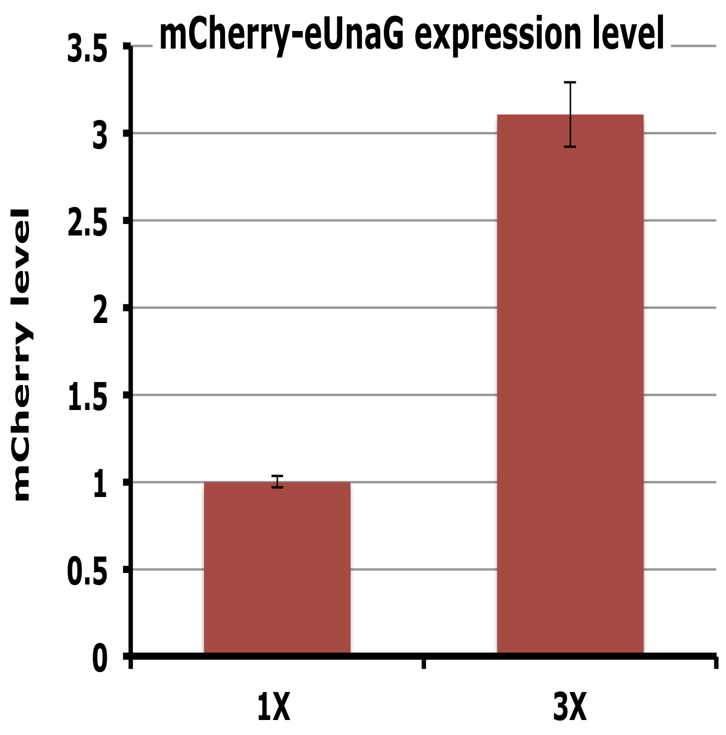

**S8. Time course and BR dosage dependent intracellular eUnaG reporter signal.**

S2R+ cells expressing mCherry-eUnaG sensor are incubated with BR (425nM, 850nM, 1700nM, 3400nM, 6800nM) for 15, 30, 60 and 120 minutes, followed by FACS analysis. Within 15 minutes saturated BR influx could be reached. However, for cells treated with lower BR concentration, it took more than 15 minutes to reach equilibrated BR influx.

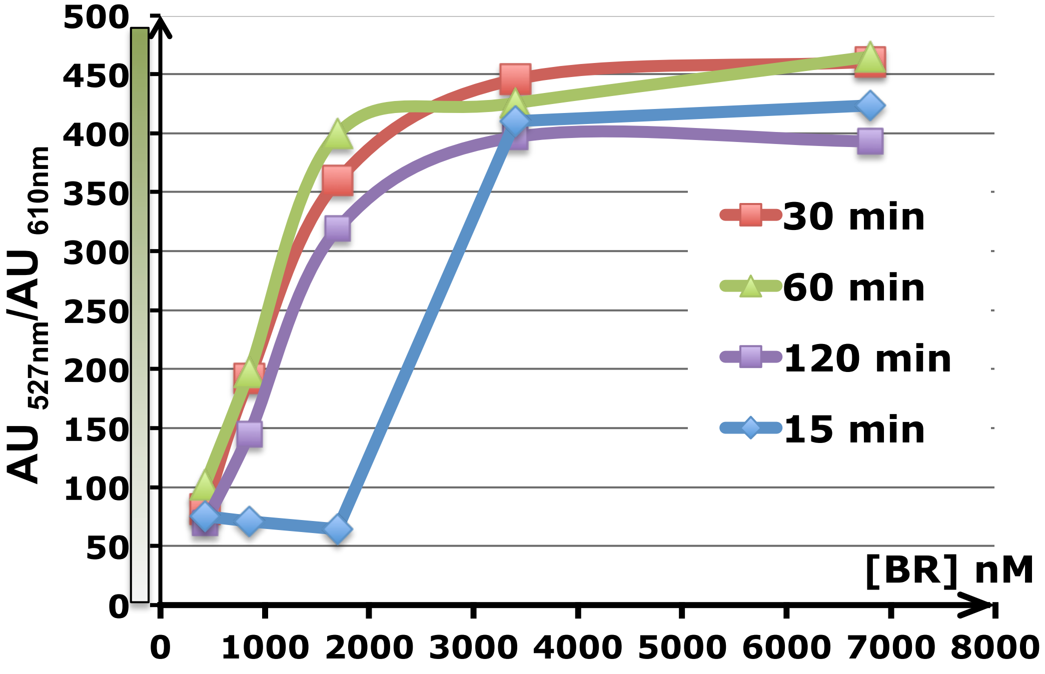

**S9. BR dose-response curve of mCherry-eUnaG signal in Kc167 cell line is different from the curve in S2R+ cell line.**

Cells expressing mCherry-eUnaG sensor were incubated with BR (15nM, 212.5nM, 425nM, 850nM, 1700 nM, 3400 nM, 6800 nM) for 60 minutes, followed by FACS analysis. However, Kc167 cells have low BR signal, indicating a different BR transport property as compared to S2R+ cells.

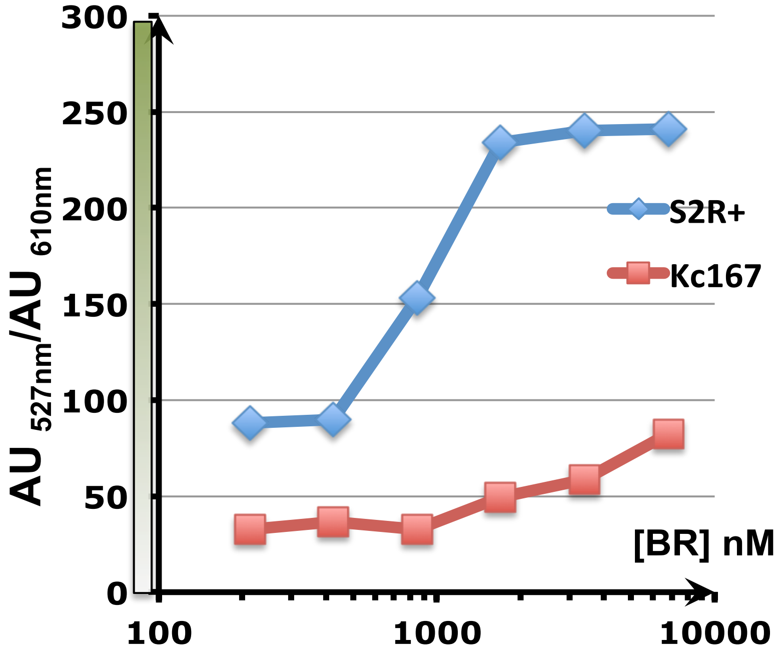

**S10. RNAi constructs.**
All RNAi constructs were designed and provided by the DRSC facility at Harvard Medical School. Detailed sequence information of each amplicon can be found at http://www.flyrnai.org/.

For amplicons that were shown in the main text Fig-4:
MRP1: Amplicon Sequence ID 264320; 0 off-target predicted.
*l*(2)03659_1: Amplicon Sequence ID DRSC07269; 0 off-target predicted.
*l*(2)03659_2: Amplicon Sequence ID DRSC07638; 0 off-target predicted.
*l*(2)03659_4: Amplicon Sequence ID DRSC34566; 0 off-target predicted
GFP: DRSC control amplicon
LacZ: DRSC control amplicon

**References:**


1. Kumagai, A. et al. A bilirubin-inducible fluorescent protein from eel muscle. *Cell* **153**, 1602-1611 (2013).
2. MacKerell, A.D. et al. All-atom empirical potential for molecular modeling and dynamics studies of proteins. *J Phys Chem B* **102**, 3586-3616 (1998).
3. Best, R.B. et al. Optimization of the additive CHARMM all-atom protein force field targeting improved sampling of the backbone phi, psi and side-chain chi(1) and chi(2) dihedral angles. *J Chem Theory Comput* **8**, 3257-3273 (2012).
4. Jorgensen, W.L., Chandrasekhar, J., Madura, J.D., Impey, R.W. & Klein, M.L. Comparison of simple potential functions for simulating liquid water. *Journal of Chemical Physics* **79**, 926-935 (1983).
5. Vanommeslaeghe, K. & MacKerell, A.D., Jr. Automation of the CHARMM General Force Field (CGenFF) I: bond perception and atom typing. *J Chem Inf Model* **52**, 3144-3154 (2012).
6. Essmann, U. et al. A smooth particle mesh Ewald method. *Journal of Chemical Physics* **103**, 8577-8593 (1995).
7. Ryckaert, J.P., Cicotti, G. & Berendsen, H.J.C. Numerical Integration of the Cartesian Equations of Motion of a System with Constraints: Molecular Dynamics of n-Alkanes. *Journal of Computational Physics* **23**, 327-341 (1977).
8. Miyamoto, S. & Kollman, P.A. Settle: An analytical version of the SHAKE and RATTLE algorithm for rigid water models. *Journal of Computational Chemistry* **13**, 952-962 (1992).
9. Brooks, B.R. et al. CHARMM: the biomolecular simulation program. *J Comput Chem* **30**, 1545-1614 (2009).
10. Jo, S., Kim, T., Iyer, V.G. & Im, W. CHARMM-GUI: a web-based graphical user interface for CHARMM. *J Comput Chem* **29**, 1859-1865 (2008).
11. Phillips, J.C. et al. Scalable molecular dynamics with NAMD. *J Comput Chem* **26**, 1781-1802 (2005).
12. Kirk, W.R., Kurian, E. & Prendergast, F.G. Characterization of the sources of protein-ligand affinity: 1-sulfonato-8-(1')anilinonaphthalene binding to intestinal fatty acid binding protein. *Biophys J* **70**, 69-83 (1996).
